# Complete mitochondrial genome of *Mukaria splendida* Distant (Hemiptera: Cicadellidae: Deltocephalinae: Mukariini) and phylogenetic analysis

**DOI:** 10.1080/23802359.2021.1875925

**Published:** 2021-02-17

**Authors:** Hui-ying Yang, Ren-huai Dai

**Affiliations:** a Institute of Entomology, Guizhou University, Guiyang, P.R. China; b The Provincial Special Key Laboratory for Development and Utilization of Insect Resources, Guizhou University, Guiyang, P.R. China

**Keywords:** Mitochondrial genome, *Mukaria splendida*, Deltocephalinae, molecular phylogeny

## Abstract

The complete mitochondrial genome (mitogenome) of *Mukaria splendida* Distant, 1908 (Hemiptera: Cicadellidae: Deltocephalinae) was first reported in Mukariini. The length of this mitogenome is 16,711 bp, which has an A + T content of 79% (A = 44.5%, T = 34.5%, G = 8.8%, C = 12.2%). A total of 37 genes were annotated [13 protein-coding genes (PCGs), 22 transfer RNA genes (tRNA), and 2 ribosomal RNA genes (rRNA)]. Among the 37 genes, 4 protein coding genes (*ND1*, *ND4*, *ND4L*, *ND5*), 8 tRNA genes (*trnQ*, *trnC*, *trnY*, *trnF*, *trnH*, *trnP*, *trnL2*, *trnV*), and 2 rRNA (*12S* rRNA, *16S* rRNA) were encoded by N chain, and the remaining genes were encoded by J chain. Overall, there were 14 gene overlaps and 9 gene gaps in the mitochondrial genome of this species. All PCGs were started with ATD (ATA/ATT/ATG), and stopped with TAR, except ATP6, which ends with single T. The phylogenetic analysis confirms that *M. splendida* clustered with other Deltocephalinae species.

The genus *Mukaria* was established with Specimens *penthimioides distance*, 1908 from Sri Lankamukaria (Distant, 1908), which belongs to Cicadellidae, Deltocephalinae, and Mukariini. Up to now, only 14 species of this genus have been recorded in the world and 10 species in China, mainly distributed in the Oriental and Palearctic regions (Yang et al. 2016). Insects of this group are important pests of bamboo (Yao et al. 2019). Most of them are similar in shape, medium in size and mostly black with glossy in color. The early literature description is simple and the identification characteristic figure is incomplete or unclear, so it is difficult to identify it accurately. The mitochondrial genome was sequenced and analyzed for its structure and phylogeny of *Mukaria splendida* (Distant 1908) provides basic data for species identification, population genetics, and evolution of the Cicadellidae.

Total genome DNA was extracted from male adult of *M. splendida* using DNeasy Blood & Tissue Kit (Qiagen), samples were collected from Yuanyang County, Yunnan Province, China (102°50′44″E, 23°13′15″ N) in August 2017. Samples and genome DNA are deposited in the Institute of Entomology, Guizhou University, Guiyang, China (GUGC-IDT-00190). Sequences were sequenced by Berry Genomics on a HiSeq 2500 platform (Illumina) with 6 Gb clean data. Mitogenome was assembled using Geneious Primer (v. 2019.2.1) under reference sequence (*Nephotettix cincticeps*, GenBank No: NC_026977).

The complete mitogenome of *M. splendida* is 16,711 bp in length (GenBank No: MG813485), containing 13 protein-coding genes (PCGs), 22 transfer RNA genes (tRNAs), 2 ribosomal RNA genes (rRNAs), and 1 large noncoding region (Control region). In general, the *M. splendida* mitogenome showed obvious AT bias, which has high A + T content of 79% (A = 44.5%, T = 34.5%, G = 8.8%, C = 12.2%). The AT-skew and GC-skew are positive (0.126) and negative (−0.164). All PCGs were started with standard starting codon (ATA, ATC, ATT, ATG), and stopped with TAR (TAA/TAG), except *ATP6*, which stopped with single T. The length of 22 tRNAs was 1432 bp, of which the shortest was *trnL1* (60 bp), and the longest was *trnK* (71 bp). Except for the secondary structure of *trnS1* (AGN) lacks DHU arm, other 21 tRNAs was typical clover structure. Among the two rRNA genes, the *16S*rRNA gene is 1205 bp in size, which was located between *trnL2* and *trnV*, and the *12S*rRNA gene is 759 bp in size, located between *trnV* gene and control region. There are 14 overlapping regions in *M. splendida*'s mitochondrial genome, with a total of 95 bp. The longest overlapping region with a length of 26 bp is between *trnH* and *ND4*, and it has 9 gene spacer regions, a total of 47 bp, of which the longest interval with a length of 19 bp is between *trnY* and *COX1*.

The phylogenetic relationships were analyzed based on the concatenated nucleotide sequences of 13 PCGs and 2 rRNAs from 46 Membracoidea species (41 Cicadellidae species and 5 Membracidae species) and two outgroups (Cicadidae). Each PCG and rRNA sequence was aligned using program of MACSE (Ranwez et al. 2011) and MAFFT (Katoh et al. 2005), respectively. And aligned sequences were eliminated using Gblocks 9.1 b (Talavera and Castresana 2007). The phylogenetic relationships of *M. splendida* were reconstructed with IQ-TREE using an ultrafast bootstrap approximation approach with 50,000 replicates (Figure 1). The phylogenetic analysis confirms that *M. splendida* clustered with other Deltocephalinae species. Up to now, not a lot of molecular studies have been reported with Mukariini, and we hope that our data can be useful for further study.

**Figure 1.**
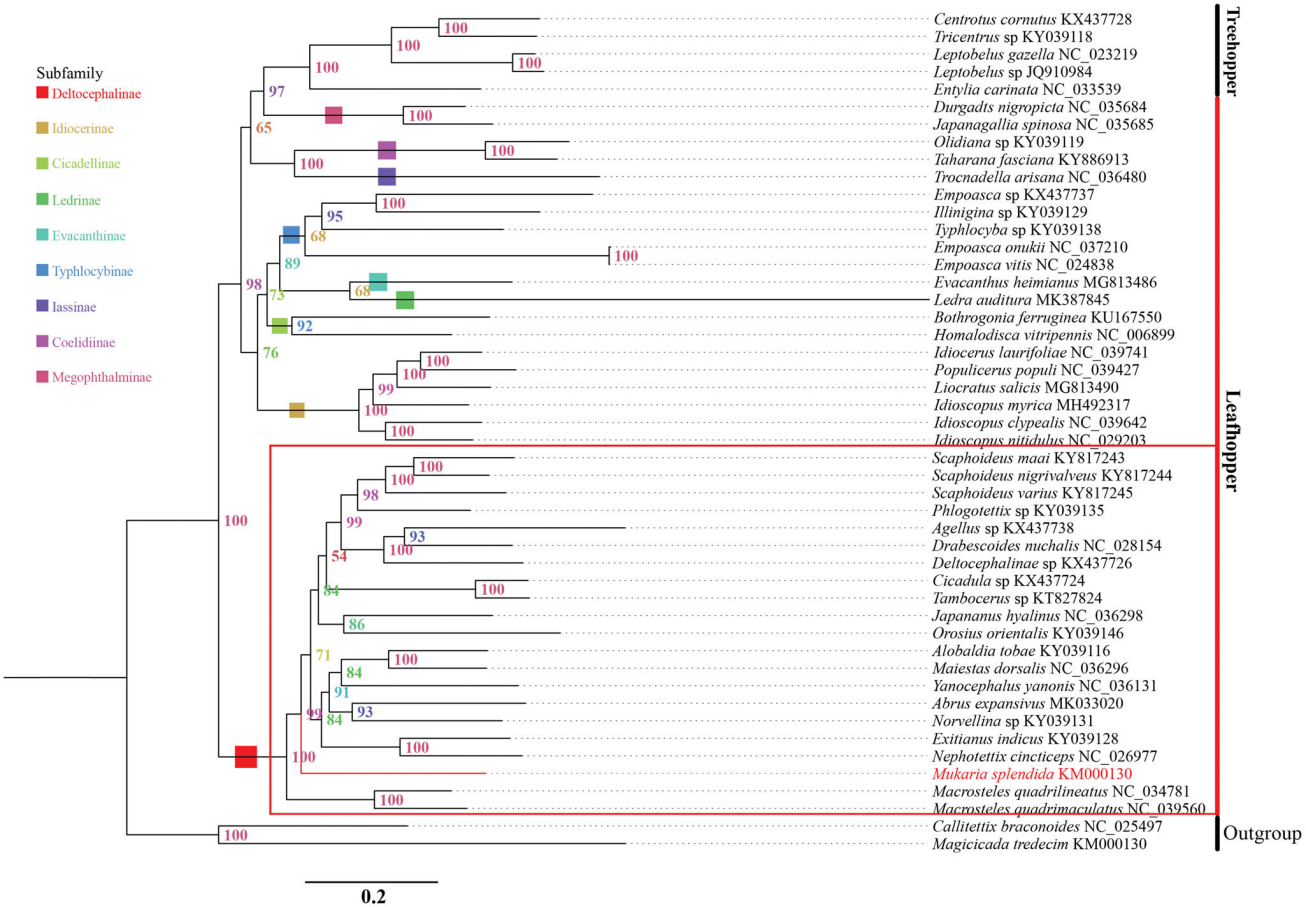
Phylogenetic relationships of the Cicadellidae based on the nucleotide sequences of the 13 PCGs and 2 rRNAs, using closely related species of the Cicadellidae.

## Data Availability

Mitogenome data supporting this study are openly available in GenBank at: https://www.ncbi.nlm.nih.gov/nuccore/MG813485. Associated BioProject, SRA, and BioSample accession numbers are https://www.ncbi.nlm.nih.gov/bioproject/PRJNA 674714, https://www.ncbi.nlm.nih.gov/sra/SRR 12989394, and https://www.ncbi.nlm.nih.gov/biosample/SAMN 16673172, respectively.
